# Protein kinase G I alpha activates an anti-remodeling signaling pathway in the heart via an interaction with the MAPKKK mixed lineage kinase 3

**DOI:** 10.1186/1471-2210-11-S1-P9

**Published:** 2011-08-01

**Authors:** Robert M Blanton, Eiki Takimoto, Angela Lane, Mark J Aronovitz, Richard H Karas, David A Kass, Michael E Mendelsohn

**Affiliations:** 1 Molecular Cardiology Research Institute; Tufts Medical Center, Boston, MA, USA; 2 Division of Cardiology, Johns Hopkins Medical Institutes; Baltimore, MD, USA; 3 Present address, Merck Research Laboratories, Rahway, NJ, USA

## Background

cGMP signaling inhibits pathologic cardiac hypertrophy and remodeling in vivo. In prior studies, we explored the role of the cGMP-dependent protein kinase I alpha (PKGIα) in regulating cardiac remodeling by studying mice with mutations in the PKGIα leucine zipper (LZ) domain [1], in which PKGIα kinase activity is retained but LZ-mediated protein-protein interactions are abolished. These mice (termed leucine zipper mutant, or LZM, mice) develop accelerated contractile dysfunction, and increased hypertrophy and mortality in response to LV pressure overload induced by Transaortic constriction, (TAC) [2], supporting a critical role for PKGIα in inhibiting remodeling in vivo. The specific myocardial signaling pathways regulated through PKGIα LZ-mediated interactions remain unknown. Cardiac JNK activation is required for normal LV compensation to TAC, and in vitro studies support a role of PKGIα in activating JNK in the cardiac myocyte. We hypothesized that PKGIα inhibits TAC-induced remodeling through activation of JNK in the heart. To test this, we explored JNK activation in LZM mouse hearts in response to various durations of TAC.

## Results

JNK activation (phosphorylation) increased from 1.0 ± 0.3 ADUs in WT sham hearts to 3.5 ± 0.6 ADUs in WT 48 hour TAC hearts (p<0.001). However, JNK phosphorylation did not increase significantly in LZM hearts post TAC (0.5 ± 0.1 ADU in LZM sham hearts vs 1.3 ± 0.2 ADU in LZM 48 hour TAC hearts, p=ns LZM sham vs LZM TAC; p<0.001 WT TAC vs LZM TAC). Similar trends in JNK phosphorylation were observed at 24 hours post-TAC and 7 days post-TAC. Loss of TAC-induced JNK phosphorylation in LZM mice was accompanied by decreased TAC-induced activation of MAP Kinase Kinase 4 (MKK4) compared with WT controls at 48 hours post-TAC.

We next explored upstream JNK regulators as potential novel PKGIα interacting molecules. In GST pulldown experiments with cardiac lysates from WT or LZM mice, the MAPKKK Mixed Lineage Kinase 3 (MLK3) bound to the WT PKGIα LZ domain, but not the mutant PKGIα LZ domain, supporting complex formation between PKGIα LZ and MLK3. Immunoprecipitation studies with WT cardiac lysates confirmed an interaction between these two signaling molecules. We next tested whether MLK3 is required for cGMP-PKGI-dependent JNK activation in cultured adult mouse cardiac myocytes (CM) following siRNA knockdown of MLK3. MLK3 knockdown blocked 8-Bromo-cGMP-stimulated JNK activation, while control siRNA had no effect on 8-Bromo-cGMP-stimulated JNK activation.

## Conclusion

These findings support that the cGMP pathway recruits the JNK signaling pathway by complex formation between PKGIα and MLK3, leading to activation of MKK4 and JNK in the heart. These findings define a novel mechanism by which cGMP and PKGIα inhibit pathological remodeling in vivo, and identify MLK3 as a potentially important therapeutic target in heart failure.

## References

[B1] Blanton RM Aronovitz M Dabreo A Karas R Mendelsohn M Genetic evidence for a central role of cGMP-dependent Protein Kinase I alpha in regulating cardiac hypertrophy in vivo Circulation 2007 116 II-213 II-214

[B2] Michael SK Surks HK Wang Y Zhu Y Blanton R Jamnongjit M Aronovitz M Baur W Ohtani K Wilkerson MK Bonev AD Nelson MT Karas RH Mendelsohn ME High blood pressure arising from a defect in vascular function Proc Nat Acad Sci USA 2008 105 6702 6707 10.1073/pnas.0802128105 18448676 PMC2373316

